# Dynamics of Platelet Counts in Major Trauma: The Impact of Haemostatic Resuscitation and Effects of Platelet Transfusion—A Sub-Study of the Randomized Controlled RETIC Trial

**DOI:** 10.3390/jcm9082420

**Published:** 2020-07-28

**Authors:** Helmuth Tauber, Nicole Innerhofer, Daniel von Langen, Mathias Ströhle, Dietmar Fries, Markus Mittermayr, Tobias Hell, Elgar Oswald, Petra Innerhofer

**Affiliations:** 1 Department of Anesthesiology and Intensive Care Medicine, Medical University of Innsbruck, 6020 Innsbruck, Austria; helmuth.tauber@tirol-kliniken.at (H.T.); daniel.von-langen@tirol-kliniken.at (D.v.L.); markus.mittermayr@i-med.ac.at (M.M.); elgar.oswald@tirol-kliniken.at (E.O.); petra.innerhofer@i-med.ac.at (P.I.); 2 Department of General and Surgical Intensive Care Medicine, Medical University of Innsbruck, 6020 Innsbruck, Austria; mathias.stroehle@tirol-kliniken.at (M.S.); dietmar.fries@i-med.ac.at (D.F.); 3 Department of Mathematics, Faculty of Mathematics, Computer Science and Physics, University of Innsbruck, 6020 Innsbruck, Austria; tobias.hell@uibk.ac.at

**Keywords:** blood coagulation disorders, fibrinogen concentrate, plasma, platelet transfusion, thrombelastography

## Abstract

Although platelets play a central role in haemostasis, the dynamics of platelet counts during haemostatic resuscitation, the response to platelet transfusion, and effects on clinical outcome are poorly described for trauma patients. As a sub-study of the already published randomized controlled RETIC Study “Reversal of Trauma-induced Coagulopathy using First-line Coagulation Factor Concentrates or Fresh-Frozen Plasma” trial, we here analysed whether the type of first-line haemostatic resuscitation influences the frequency of platelet transfusion and determined the effects of platelet transfusion in coagulopathic patients with major trauma. Patients randomly received first-line plasma (FFP) or coagulation factor concentrates (CFC), mainly fibrinogen concentrate. In both groups, platelets were transfused to maintain platelet counts between 50 and 100 × 10^9^/L. Transfusion rates were significantly higher in the FFP (n = 44) vs. CFC (n = 50) group (FFP 47.7% vs. CFC 26%); *p* = 0.0335. Logistic regression analysis adjusted for the stratification variables injury severity score (ISS) and brain injury confirmed that first-line FFP therapy increases the odds for platelet transfusion (odds ratio (OR) 5.79 (1.89 to 20.62), *p* = 0.0036) and this effect was larger than a 16-point increase in ISS (OR 4.33 (2.17 to 9.74), *p* = 0.0001). In conclusion, early fibrinogen supplementation exerted a platelet-saving effect while platelet transfusions did not substantially improve platelet count and might contribute to poor clinical outcome.

## 1. Introduction

The current European Guidelines on the management of trauma-related bleeding recommend early transfusion of plasma and red blood cells at least at ratios of 1:2 or administration of fibrinogen concentrate along with red blood cell transfusion. In addition, platelets (PLT) shall be administered to maintain a PLT count above 50 to 100 × 10^9^/L, although the evidence for PLT transfusion at certain thresholds is weak for patients with multiple trauma, and the effect of PLT transfusion is controversial [1]. Several studies investigated the prevalence of thrombocytopenia at hospital admission and its relation to severity of injury, morbidity, and mortality [2,3,4,5]. However, PLT counts during haemostatic resuscitation and especially following transfusion of PLT are rarely reported in the literature [6,7,8,9]. As assumed by the authors of a large observational trial, the progression of PLT count after hospital admission may be influenced by initial bleeding management [6], which most frequently consists of transfusion of fresh-frozen plasma (FFP) and red blood cell concentrates (RBC) at various ratios [1]. Interestingly, some data indicate that the need for PLT transfusion can be reduced with use of coagulation factor concentrates (CFC) [8,9,10]. However, in those studies. haemostatic therapy followed the decision of the anaesthetist and was not controlled.

In the RETIC study, severely injured patients randomly received either FFP or CFC for correction of poor fibrin polymerization and/or prolonged initiation of coagulation. The protocol started with randomization after hospital admission and lasted up to 24 h after intensive care unit (ICU) admission. More than 80% of patients in both groups needed immediate surgery and received comparable amounts of balanced crystalloid and colloid solutions. Transfusion thresholds for RBC and PLT were identical for both groups to maintain haemoglobin between 8 and 10 g/dL and PLT count between 50 and 100 × 10^9^/L [11]. Therefore, a direct comparison of two different haemostatic therapies concerning dynamics of PLT count and need for PLT transfusion was feasible. We hypothesized that the need for PLT transfusion correlates with severity of injury but is influenced by the type of haemostatic resuscitation. We further hypothesized that PLT transfusion has no enduring effect on PLT numbers and might influence clinical outcome.

## 2. Experimental Section

The study protocol was approved by the Ethical Committee of the Medical University of Innsbruck, Austria (Study code: UN4497), Chairperson Prof Dr. P. Lukas on October 2011. The study is registered with ClinTrials.gov NCT01545635 and EudraCT 2011-004139-29.

The primary endpoint of the RETIC Study “Reversal of Trauma-induced Coagulopathy using First-line Coagulation Factor Concentrates or Fresh-Frozen Plasma” was to detect a difference in MOF (multiple organ failure) in patients receiving FFP or CFC; the protocol and first results have been published [11]. In brief, patients exhibiting an injury severity score (ISS) > 15, clinical signs or risk for substantial haemorrhage and thromboelastometry (ROTEM, TEM International)-diagnosed poor fibrin polymerisation (FibTEM A10 < 9 mm), and/or prolonged initiation of coagulation (ExTEM CT > 90 s) were randomly treated with either FFP or fibrinogen concentrate. A 4-factor prothrombin complex concentrate and FXIII concentrate were also used in selected cases. Rescue therapy was initiated in a crossover fashion if two-times study drug administration did not correct coagulopathy and diffuse or massive bleeding persisted. After each study drug administration, the ROTEM was repeated bedside, and samples were sent to the central laboratory for performing blood cell count and plasmatic coagulation tests. Platelet concentrates (one apheresis concentrate or six pooled platelet concentrates, platelet count at least 3 × 10^11^/L) were administered to maintain a platelet count of 50 to 100 × 10^9^/L and/or if clot firmness remained poor (ExTEM A10 < 35 mm) albeit with sufficient fibrinogen polymerization (FIBTEM A10 > 15 mm). RBC were transfused to maintain haemoglobin between 8 and 10 g/dL. The study protocol started with randomization and was continued until 24 h after ICU admission. The Ethics Committee waived the need for initial informed consent, but written informed consent was obtained as soon as the patient regained legal capacity. Pre-planned interim analysis after inclusion of 100 patients demanded early study termination because predefined stopping rules were met.

Baseline characteristics and ISS were balanced between the FFP and CFC groups and the frequency of immediate surgery was comparable. The majority of RETIC patients received FFP or CFC during treatment in the emergency department and immediate surgery, before admission to ICU. Patients receiving first-line FFP therapy had higher rates of MOF than did patients treated with CFC, and logistic regression adjusted for the stratification factors ISS and brain injury confirmed that FFP transfusion significantly increased the risk for MOF (odds ratio (OR) 3.13 (1.19 to 8.88), *p* = 0.025). In addition, 52.3% patients in the FFP group showed no correction of coagulopathy after double-dose FFP administration and thus received CFC, while only 4% of CFC patients also required FFP. Patients with first-line FFP therapy had significantly higher bleeding scores than those receiving CFC, time to haemostasis was significantly longer, and the frequency of massive transfusion, defined as RBC ≥ 10/24 h, was significantly higher. [11].

### 2.1. The Present Sub-Study

As a pre-planned secondary endpoint of the RETIC trial, we here analyse the development of PLT counts in patients treated with FFP or CFC, and in patients receiving PLT transfusion or not. We also explore the association between PLT counts and achieved levels of fibrinogen (Clauss method) and clot firmness (ROTEM EXTEM MCF) because FFP and CFC treatment affect these parameters differently. According to the RETIC protocol, blood samples for various coagulation measurements including PLT count were drawn at several time points. We here analyse measurements at admission to the emergency department, at randomization, after first study drug administration, on admission to ICU, and 24 and 48 h thereafter.

### 2.2. Outcomes

We assessed the effect of haemostatic therapy on the need for PLT transfusion and analysed patients’ characteristics stratified for PLT transfusion in the entire study population and an ISS-matched study population. We explored the progression of PLT counts, fibrinogen concentrations, and clot firmness in the matched population stratified for use of FFP or CFC and PLT transfusion. We also analysed changes in PLT counts in the full study population, and clinical outcome of the entire and the ISS-matched population factorized by PLT transfusion.

### 2.3. Statistical Analysis

In this work, we performed pre-planned descriptive and exploratory post-hoc analyses. A mathematician not involved in our trial procedures or patient assessment (TH) conducted the statistical analyses using R, version 3.4.1. All statistical assessments were two-sided and a significance level of 5% was used. We applied the Wilcoxon rank sum test for continuous variables and Fisher’s exact test for binary variables to assess differences between treatment groups as well as between patients with and without PLT transfusion. We present continuous data as median (IQR) and categorical variables as frequencies (%). We show effect size and precision with estimated median differences between groups for continuous data and odds ratios (OR) for binary variables, with 95% confidence intervals (CIs). We performed logistic regression analysis for platelet transfusion adjusted for ISS and brain injury, as those factors were confounders also used for stratification for patient randomization. As the occurrence of PLT transfusion is clearly confounded by the severity of injury, we conducted exact matching with respect to the ISS. We illustrate the matching by providing propensity scores and differences in baseline characteristics before and after matching.

For the matched population factorized by treatment and PLT transfusion, we provide the progression of PLT, fibrinogen, and clot firmness descriptively using the course of the median with corresponding 95% CIs. At six measurement time points we assess significant differences after applying a respective Bonferroni correction.

## 3. Results

The RETIC study enrolled 100 trauma patients with trauma-induced coagulopathy (TIC) as diagnosed with rotational thromboelastometry (ROTEM). After randomization, six patients dropped out. Thus, 94 patients (FFP group n = 44; CFC group n = 50) made up the modified intent-to-treat population [11].

**Figure 1:**
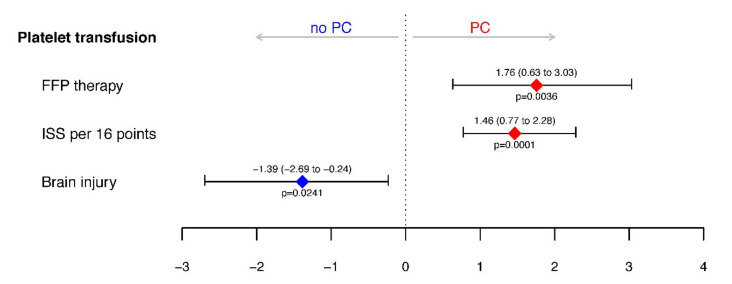
Effect of fresh-frozen plasma (FFP) therapy on the probability of platelet transfusion (PC, platelet concentrate) adjusted for stratification factors injury severity score (ISS) and brain injury. Depicted are the log odds ratios with 95% confidence intervals (CIs) retrieved from logistic regression for platelet transfusion during emergency treatment until 48 h after admission to the ICU. Corresponding adjusted odds ratios are 5.79 (1.89–20.62) for FFP therapy, 4.33 (2.17–9.74) for ISS, and 0.25 (0.07–0.78) for brain injury.

At admission, study patients had a PLT count of 182.5 × 10^9^/L (154.25 to 216.25) and 19 (20.21%) of the 94 patients showed PLT numbers below the lower normal value of 150 × 10^9^/L. During the observation period, 34 (36.2%) of the 94 study patients received at least 1 U, median 2 U (1 to 3), of PLT concentrates. Comparing the PLT transfusion rates in patients of the FFP or CFC group we found a significantly higher transfusion rate in the FFP than in the CFC arm (21/44 (47.7%) vs. 13/50 (26%)); OR 2.57 (1.01 to 6.79), *p* = 0.034. Logistic regression analysis adjusted for the stratification variables ISS and brain injury confirmed that first-line FFP therapy increases the odds for platelet transfusion (OR 5.79 (1.89 to 20.62), *p* = 0.0036) and this effect was larger than a 16-point increase in ISS (OR 4.33 (2.17 to 9.74), *p* = 0.0001) (Figure 1).

By analysing the entire RETIC population stratified by PLT transfusion yes or no, we found that patients after receiving PLT transfusion had a significantly higher ISS, were admitted with lower blood pressure, haemoglobin, fibrinogen, and higher international normalised ratio (INR) values, but comparable PLT counts than those who did not (Table 1).

**Figure 2:**
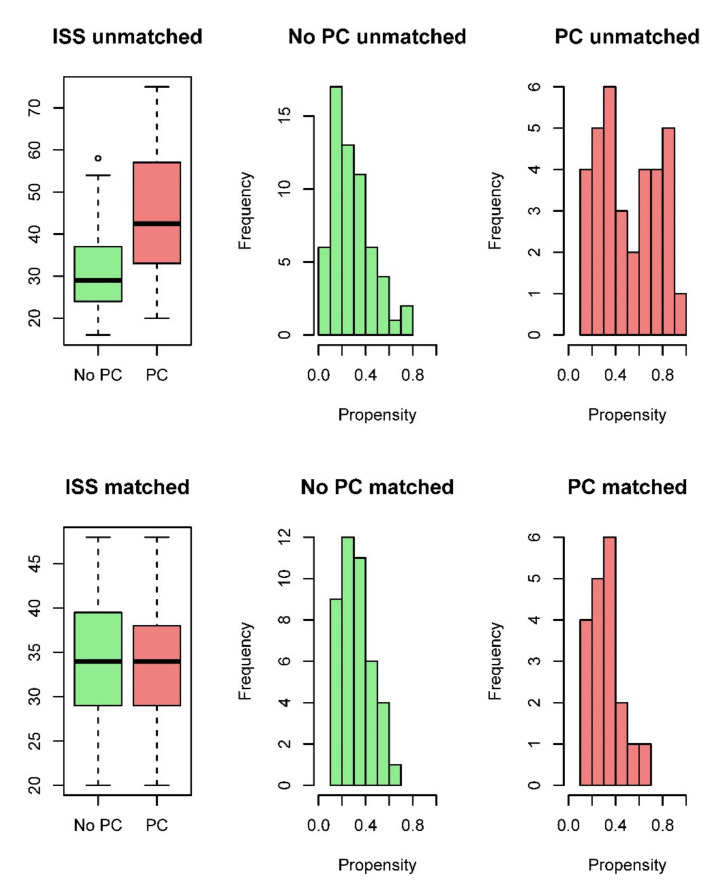
ISS (box plots) and distribution of the propensity score for receiving platelet concentrates (PC) (depicted by frequencies) before (first row) and after matching (second row).

Therefore, we next performed exact ISS matching. Figure 2 demonstrates that the ISS and the distribution of the propensity scores for PLT transfusion differed before ISS matching, but were comparable after matching. The ISS-matched population (n = 62) showed no statistically significant differences in baseline characteristics, (Table 1), indicating that adequate balance was achieved.

**Figure 3:**
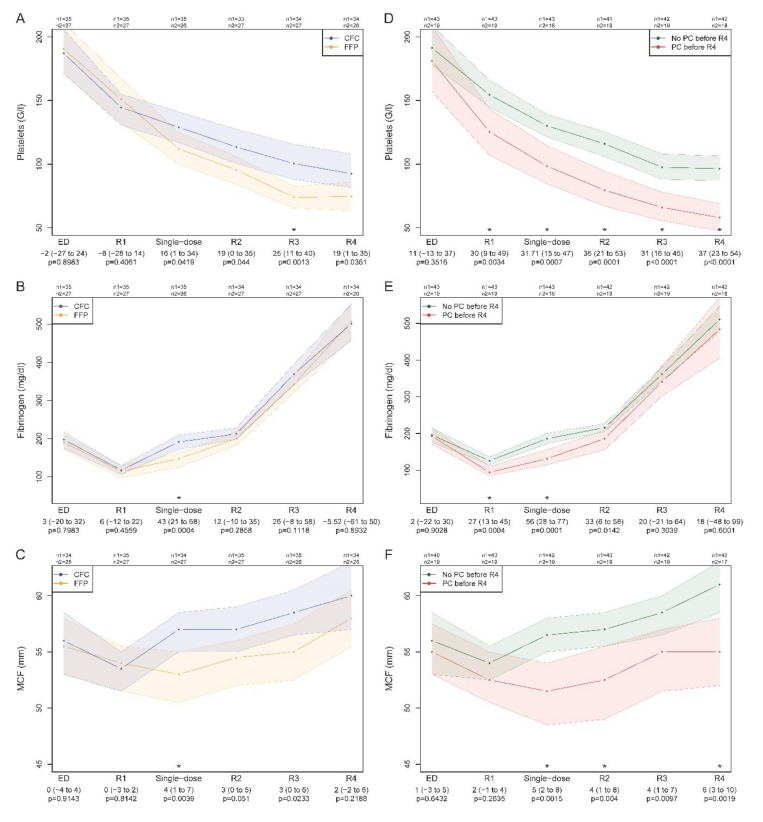
Development of platelet count, fibrinogen concentration, and maximum clot firmness (MCF) in the matched population stratified for haemostatic therapy (**A**–**C**) using first-line transfusion of fresh-frozen plasma (FFP) or administration of coagulation factor concentrates (CFC), and stratified for platelet transfusion (PC, platelet concentrate) yes or no (**D**–**F**). ED, admission to emergency department; R1 at randomization before therapy; single-dose, after first study drug administration; R2, admission to ICU; R3, after 24 h in ICU; R4, after 48 h in ICU. The *p* values correspond to the Wilcoxon rank sum test; confidence intervals are provided for the estimated median difference. Asterisks mark a significant difference at the respective time point after Bonferroni correction.

In this ISS-matched population, 19 (30.6%) of the 62 patients received 2 U (1 to 3) of PLT concentrates; as in the entire population, the transfusion rate was significantly higher in the FFP (14/27 (51.9%) vs. 5/35 (14.3%)) than in the CFC arm; OR 6.25 (1.69 to 27.09), *p* = 0.0022. Because the first-line therapy with FFP or CFC differed mainly in early achieved levels of fibrinogen and clot firmness, we analysed the progression of these variables in relation to PLT count (Figure 3).

The intrinsic effects of FFP and CFC administration on PLT numbers (Figure 3A–C) are best depicted at the interval between R1 and the measurement point single-dose, which shows achieved values after the first study drug administration. Single-dose CFC (mainly fibrinogen concentrate) slowed the drop of platelets (Figure 3A) simultaneously with the achievement of fibrinogen concentration towards 200 mg/dl (Figure 3B) and improvement of clot strength (Figure 3C). After single-dose FFP administration, platelets dropped sharply, fibrinogen remained below normal levels, and clot firmness decreased. Comparing these variables in patients receiving PLT or not (Figure 3D–F), we found comparable PLT counts at admission, those who received PLT exhibited an early steep decline in PLT counts, (Figure 3D), had significantly lower fibrinogen concentrations during the early course of haemostatic resuscitation (Figure 3E), and lower clot firmness (Figure 3F) and PLT numbers throughout the observation period.

Platelet transfusion did not limit blood loss as reflected by significantly increased transfusion of RBC (12 U (9–14) vs. 3 U (2–5); estimated difference −8 (−9 to −6), *p* < 0.0001) and frequency of massive transfusion (RBC ≥ 10 U/24 h), which occurred in 12/19 (63.2%) patients receiving PLT vs. 1/43 (2.3%) patients without PLT transfusion; OR 64.65 (7.65 to 3079.32), *p* < 0.0001.

**Figure 4:**
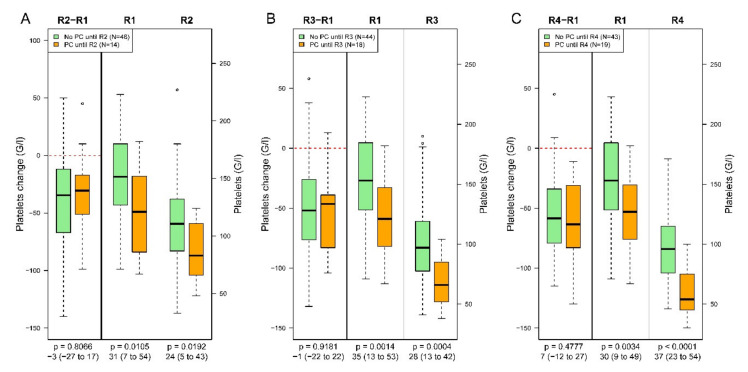
Change in platelet count between inclusion (R1) and measurement points admission ICU (R2, panel (**A**)), 24 h ICU (R3, panel (**B**)) and 48 h ICU (R4, panel (**C**)) for patients with and without platelet transfusion (PC). Data refer to the full study population. The *p* values correspond to the Wilcoxon rank sum test; confidence intervals are provided for the estimated median difference.

Analysing the data of all study patients regarding the change in PLT numbers following PLT transfusion, we found that PLT continuously declined and the change from baseline to the time point after transfusion was similar, regardless of whether patients had received PLT or not (Figure 4).

PLT transfusion was associated with posttraumatic organ failure and mortality (Table 2). Notably, in the unmatched population, seven (7.4%) of the 94 study patients did not survive, all seven of whom had received PLT. In the ISS-matched population PLT transfusion was associated with coagulation and renal failure, as assessed by sequential organ failure assessment (SOFA) score. Patients receiving PLT showed significantly higher rates of MOF, hemofiltration, sepsis, venous thrombosis, and mortality. They also had fewer ventilator-, ICU- and hospital-free days. However, these latter differences were not statistically significant.

## 4. Discussion

Platelets (PLT) play a central role in haemostasis, and their role in modulating the inflammatory response is increasingly recognized [12,13,14,15]. PLT and fibrinogen are important substrates needed for clot formation. Thus, PLT are an important factor in avoiding early death from exsanguination in major trauma. PLT may also be important in the later phase of trauma, where patients are at risk for developing multiple-organ failure (MOF), which is characterized by a misbalanced inflammatory response [15].

We here investigated the course of PLT count in patients with major trauma, bleeding signs, and ROTEM-diagnosed coagulopathy, who randomly received either first-line FFP or CFC. The main findings are (i) PLT drop sharply during the first hours of haemostatic resuscitation and decline continuously thereafter, (ii) early fibrinogen administration limits blood loss by improving clot strength and consequently reduces PLT transfusion rates, (iii) PLT transfusion did not substantially increase PLT count, regardless of whether patients received FFP or CFC, (iv) transfusion of PLT concentrates was associated with poor clinical outcome.

As observed in other studies, the majority of our study patients had PLT counts in the normal range at admission, but 34 (36.2%) of the 94 study patients needed PLT transfusion to maintain PLT at recommended thresholds of 50–100 × 10^9^/L [1]. The PLT transfusion rate in the FFP arm was nearly double that in the CFC arm, although platelet counts were comparable at baseline as were ISS, frequency and type of immediate surgery, and volume supply [11]. Because univariate analysis revealed that PLT transfusion is associated with ISS, blood pressure, fibrinogen, and INR at admission, we also analysed an ISS-matched sub-population showing differences in baseline variables that were statistically not significant. In this subpopulation the difference in PLT transfusion rate between the FFP and CFC group remained significantly different, patients treated with FFP had an OR of 6.25 to receive PLT transfusion as compared to CFC treatment. In addition, logistic regression analysis adjusted for ISS and brain injury (used for stratification at randomization) revealed that the use of FFP significantly increased the odds for receiving PLT transfusion. This effect was even larger than an increase in ISS of 16 points. In the ISS-matched population, PLT counts decreased sharply towards significantly lower values after first-dose FFP transfusion than after first-dose CFC, reaching levels reported by Chambers and co-workers who used FFP for haemostatic management in another observational trial also using FFP [6,16]. In both studies, a sharp decrease in PLT count was noted two hours after admission, which approximately meets our measurement point single dose. We also confirm that PLT numbers declined continuously thereafter, whereby the lowest counts occurred in the FFP group, although these patients received PLT transfusions more frequently than did those in the CFC group.

There are several reasons why PLT drop more sharply with FFP-based therapy than with use of CFC. The volume-expanding effect of FFP transfusion may be one contributing factor. Considering the results of a study investigating volume expansion after plasma transfusion, the anticipated decline in PLT would be in the range of 5% from baseline at two hours [17]. This would correspond to a decrease in PLT of 9 × 10^9^/L in our study patients. Actually, PLT declined much more than that, and the difference between treatment groups was also larger. However, the above-cited study worked with volunteers, while the RETIC study patients had coagulopathy and ongoing blood loss. We hypothesize that in injured patients, the drop in PLT predominately depends on the severity and duration of bleeding, the course of which was significantly worse in patients in the FFP group, showing significantly higher bleeding scores and prolonged bleeding as compared to that of the CFC group [11].

In trauma, fibrinogen concentrations and clot firmness are strongly associated with blood loss and even mortality. Considerable evidence exists to show that fibrinogen concentrate improves clot firmness more or the same as does PLT transfusion and also independently of PLT count [5,18,19,20,21,22,23,24]. Early correction of hypofibrinogenemia towards normal levels by administration of fibrinogen concentrate increases clot strength, which is a precondition for cessation of bleeding and, thus, attenuates the loss of PLT and the need for transfusion of PLT concentrates.

The efficacy of PLT transfusion in the perioperative setting in ICU patients and especially in trauma patients is controversial [1,25,26]. Moreover, supplying PLT concentrates is sometimes difficult for blood banks, and transfusion carries the risk of several serious side-effects. To the best of our knowledge, only a few studies have addressed PLT counts following PLT transfusion in trauma patients [7,8,9,16,27]. Unfortunately, the large observational study investigating development of PLT after hospital admission could not assess the effects of PLT transfusion [6]. We found that PLT transfusion did not substantially increase PLT counts; the change from baseline to the respective time point after PLT transfusion was comparable regardless of whether patients had received PLT transfusion or not. These results are in line with those of an observational trial using PLT transfusion at fixed ratios to RBC and FFP [7]. Kornblith and co-workers reported that early PLT transfusion (0–24 h) resulted in a minimal expected increase of PLT count, followed by a decrease below pretransfusion values; the posttransfusion increase in PLT numbers was larger with late transfusions after 48 h [27]. These results confirm our findings that PLT transfusion did not substantially increase PLT count in the time period up to 48 h at ICU. The regression model referred to the administration of 1 U PLT concentrate, which is the recommended dosage as mentioned in the European Guidelines [1]. Such dosages may be sufficient to prevent spontaneous bleeding in oncologic patients, but it might be insufficient to compensate PLT loss in the bleeding trauma patient [28]. Currently, some data suggest a reduction in early death from exsanguination with formula-driven transfusion protocols using early PLT transfusion without prior measurements of platelet numbers [29]. However, it is difficult to separate the effects of PLT transfusion from those of simultaneously administered RBC and FFP, and PLT numbers were not described in that study. Interestingly, clot firmness did not normalize in one study testing such a transfusion protocol, suggesting that PLT count did not improve sufficiently [30]. Another study found that increased PLT transfusion rates and dosages did not prevent the sharp drop in PLT within the first two hours [16].

Considering the pathophysiology of severe trauma, it is conceivable that the huge levels of adrenalin, tissue factor, von Willebrand factor, and FVIII, formation of micro-particles, histones, damage-associated molecular patterns, and several other mediators directly or indirectly activate PLT [31,32,33,34,35]. Activated PTL are rapidly removed from the circulation, which could explain why PLT count did not substantially improve after PLT transfusion. Vulliamy and co-workers reported increased length of hospital and ICU stay and an increased rate of MOF in patients receiving PLT as compared to patients treated without [7]. We also observed an association between PLT transfusion, MOF, and hemofiltration, sepsis, venous thrombosis, and in-hospital mortality, even in the smaller ISS-matched population. Interestingly, in internal patients prophylactic PLT transfusion was associated with venous thrombosis and mortality [36]. Another retrospective study found that preoperative PLT transfusion was associated with increased ICU admission rates and length of hospital stay [37]. In addition, increased mortality due to acute respiratory distress syndrome has been reported for patients undergoing orthotopic liver transplantation and receiving PLT [38].

Neither the above-cited retrospective studies nor our prospective data can prove a causal relationship between PLT transfusion and poor outcome. In severe trauma, PLT transfusion is most likely a surrogate parameter of tissue damage, pronounced blood loss, and huge transfusion requirements, both of which are associated with morbidity and mortality. However, blood loss not only depends on the severity of injury but also on coagulation management, which influences duration and amount of bleeding. Our results indicate that early fibrinogen supplementation exerts a platelet-saving effect, and thus appears to be a promising strategy to minimize development of severe thrombocytopenia in a substantial proportion of trauma patients.

Several limits of our study need to be discussed. The RETIC randomized patients to be treated with FFP or CFC and not to receive PLT transfusion or not. However, the transfusion threshold for PLT concentrates was identical in the FFP and CFC groups, as were baseline characteristics, frequency of immediate surgery, and volume supply [11]. Because patients of the FFP group had a worse bleeding situation, it was not unexpected that PLT numbers would decline more sharply than in patients of the CFC group. The new finding here reported is that the early effective correction of hypofibrinogenemia by use of fibrinogen concentrate limited further blood loss through augmenting clot strength, and early improvement of clot strength slowed the decline of PLT numbers. In addition, group assignment strongly influenced transfusion rates, however, PLT transfusion did not substantially increase PLT count in both the FFP and CFC groups. These unexpected results indicate that transfusion of PLT at recommended thresholds and dosages had no substantial effect even in patients of the CFC group showing lower bleeding scores. As the RETIC study was not designed to evaluate efficacy of PLT transfusion, these findings need to be investigated in further studies.

Exact ISS matching without considering base deficit (BD) as a surrogate parameter for haemorrhage may be questioned. However, after ISS matching, all baseline parameters including BD were comparable for patients who later received PLT or not. Because BD as well as values of pH and lactate were even numerically higher in the group receiving no PLT transfusion, we performed no further matching. Critics may believe that the study population was small. Data of 94 severely injured patients (median ISS 34) were available for analysis, this is a remarkable number as the study started in March 2012 and was prematurely terminated due to stopping rules in February 2016. Exact ISS matching reduced the number of cases but increased the significance of results. Moreover, until now, no controlled study compared the course of PLT counts and need for laboratory guided PLT transfusion in patients receiving either FFP or CFC. The observational studies of Vulliamy or Kornblith present larger populations (mean ISS of 29), however, the study periods were exceptionally long, 7 and 11 years, respectively [7,27]. In addition, those studies used fixed transfusion algorithms without any comparator. Another limit is that usually the response to PLT transfusion is evaluated by measuring the current count increment (CCI) for PLT within one hour after transfusion. We measured PLT counts at longer intervals. However, the 20 h CCI was highly correlated to the 1 h CCI in a study investigating factors influencing increments following PLT transfusion [39].

Lastly, we did not register the storage time of transfused PLT, because at our institution the storage time for PLT concentrates averages only three days.

## 5. Conclusions

In conclusion, we here found a strong association between type of haemostatic resuscitation and development of thrombocytopenia in severely injured patients. Early fibrinogen supplementation exerts a platelet-saving effect and significantly reduces the need for PLT transfusion in a substantial proportion of trauma patients. Further studies are urgently needed to enlarge our knowledge regarding the effects of PLT transfusion in trauma patients.

## Figures and Tables

**Table 1:** Characteristics of patients at admission to emergency department stratified for platelet transfusion received during haemostatic resuscitation until 48 h after ICU admission.

Demographics	Unmatched Population				Matched Population			
	No PC (n = 60)	PC (n = 34)	Estimate with 95% CI	*p* Value	No PC (n = 43)	PC (n = 19)	Estimate with 95% CI	*p* Value
Age (years)	40.5 (25.75–51.25)	47.5 (26.25–58.75)	−3 (−10 to 4)	0.3952	44 (30.5–51.5)	54 (30–62)	−8 (−18 to 3)	0.1343
Male Sex	49/60 (81.7%)	21/34 (61.8%)	0.37 (0.13 to 1.05)	0.0484	33/43 (76.7%)	13/19 (68.4%)	0.66 (0.17 to 2.69)	0.5378
Time to ED (min)	61.5 (40–90)	59 (45.25–78.25)	3.15 (−10 to 20)	0.6508	58 (40–86)	60 (42–76.5)	0 (−18 to 20)	0.9756
ISS (pts)	29 (24–36.5)	42.5 (33.25–57)	−12 (−18 to −7)	<0.0001	34 (29–39.5)	34 (29–38)	0 (−5 to 4)	0.8841
AIS brain > 2	16/60 (26.7%)	13/34 (38.2%)	1.69 (0.63 to 4.57)	0.2556	15/43 (34.9%)	6/19 (31.6%)	0.86 (0.22 to 3.08)	1
Systolic BP (mmHg)	118 (93.75–140)	90 (70.5–127.5)	20 (2 to 34)	0.0181	120 (100–135)	120 (90.5–139.5)	0 (−20 to 15)	0.7827
Heart rate (bts/min)	92.5 (80.75–108.25)	110 (85–116)	−10 (−20 to 1)	0.0688	93 (82–108.5)	100 (72–110)	4 (−10 to 17)	0.6349
Blood pH	7.33 (7.27–7.37)	7.31 (7.17–7.36)	0.04 (0 to 0.09)	0.0679	7.31 (7.26–7.38)	7.35 (7.32–7.37)	−0.03 (−0.07 to 0.02)	0.219
BD (mmol/L)	3.7 (1.23–5.62)	4.5 (3.23–9.35)	−1.8 (−3.6 to 0)	0.05	4.7 (1.85–6.15)	3.8 (2.15–4.85)	0.8 (−0.9 to 2.4)	0.2955
Lactate (mmol/L)	2.16 (1.44–2.8)	2.33 (1.8–4.86)	−0.44 (−1.22 to 0.11)	0.0875	2.44 (1.55–2.94)	1.89 (1.55–2.33)	0.33 (−0.11 to 0.78)	0.1485
Hemoglobin (g/dL)	12.2 (10.85–13.4)	10.05 (9–11.8)	2 (1 to 2.9)	0.0002	11.9 (10.6–12.9)	10.5 (9.8–12.3)	0.9 (−0.3 to 2)	0.1364
Fibrinogen (mg/dL)	189.5 (166–222)	171.5 (112.5–208.25)	30.73 (3 to 58)	0.029	205 (150–224.5)	193 (171.5–225)	2 (−22 to 30)	0.9028
Platelets (G/L)	190.5 (156.75–217.25)	166.5 (150–202.5)	17 (−2 to 36)	0.0848	189 (157.5–220.5)	180 (151–215)	11 (−13 to 37)	0.3516
PTI (%)	69 (61.75–84.25)	58.5 (44.25–68.25)	14 (7 to 21)	0.0002	69 (61.5–85)	66 (58.5–74)	5 (−3 to 13)	0.151
INR	1.2 (1.1–1.33)	1.4 (1.2–1.67)	−0.2 (−0.3 to −0.1)	0.0004	1.2 (1.1–1.35)	1.3 (1.2–1.4)	−0.1 (−0.1 to 0)	0.2553
aPTT (s)	31.5 (27.75–33.25)	37 (29.25–49)	−7 (−12 to −3)	0.0004	32 (29–33.5)	34 (28–37.5)	−2 (−5 to 2)	0.3348
ExCT (s)	54 (51–63)	64 (49–82)	−7 (−16 to 0)	0.0552	56.5 (52–67.25)	49 (47.5–64)	5 (−1 to 10)	0.0959
ExMCF (mm)	56 (50–59)	54 (49.25–57)	3 (−1 to 5)	0.1195	57 (48.75–61.25)	54 (53–57.5)	1 (−3 to 5)	0.6432
FibA10 (mm)	9 (6–11)	8 (4.25–10)	1 (0 to 3)	0.0736	9 (5.75–12.25)	9 (8–10.5)	−1 (−3 to 2)	0.6716

Inf, infinitive means >4 at sofa score. mathematical correct is infinitive if it is >4. Binary data are presented as no./total no. (%) and continuous data as medians (IQR). Estimates are odds ratios for binary variables and estimated median differences for continuous variables. Differences between patients without and with platelet transfusion assessed by Fisher’s Exact Test for binary variables and Wilcoxon Rank Sum Test for continuous variables. PC, Platelet Concentrate; ISS, Injury Severity Score; AIS, Abbreviated Injury Severity Score; BP, Blood Pressure; BD, Base Deficit; PTI, Prothrombin Time Index (Quick); INR, International Normalised Ratio; aPTT, Activated Partial Thromboplastin Time; ExCT, Coagulation Time of ExTEM Assay; ExMCF, Maximum Clot Firmness of ExTEM Assay; FibA10, Fibrin Polymerization at 10 min.

**Table 2:** Clinical outcome of patients receiving platelet transfusion or not during haemostatic resuscitation until 48 h after ICU admission.

Organ Failure (SOFA > 2)	Unmatched Population				Matched Population			
	No PC (n = 60)	PC (n = 34)	Estimate with 95% CI	*p* Value	No PC (n = 43)	PC (n = 19)	Estimate with 95% CI	*p* Value
Respiration	18/60 (30%)	18/34 (52.9%)	2.6 (1.01 to 6.86)	0.046	15/43 (34.9%)	6/19 (31.6%)	0.86 (0.22 to 3.08)	1
Coagulation	4/60 (6.7%)	20/34 (58.8%)	19.15 (5.32–89.81)	<0.0001	2/43 (4.7%)	13/19 (68.4%)	39.99 (6.82–447.59)	<0.0001
Liver	0/60 (0%)	6/34 (17.6%)	Inf (2.3 to Inf)	0.0017	0/43 (0%)	2/19 (10.5%)	Inf (0.43 to Inf)	0.0904
Cardiovascular	48/60 (80%)	34/34 (100%)	Inf (1.8 to Inf)	0.0035	37/43 (86%)	19/19 (100%)	Inf (0.54 to Inf)	0.1647
Central nervous system	17/60 (28.3%)	15/34 (44.1%)	1.98 (0.75 to 5·26)	0.1737	12/43 (27.9%)	6/19 (31.6%)	1.19 (0.3 to 4.39)	0.77
Renal	1/60 (1.7%)	12/34 (3.3%)	30.99 (4.15 to 1387.39)	<0.0001	0/43 (0%)	6/19 (31.6%)	Inf (3.27 to Inf)	0.0004
Multiple organ failure	25/60 (41.7%)	29/34 (85.3%)	7.93 (2.56 to 29.96)	<0.0001	18/43 (41.9%)	15/19 (78.9%)	5.07 (1.32 to 24.56)	0.012
Sepsis	4/60 (6.7%)	12/34 (35.3%)	7.45 (1.99 to 35.14)	0.001	2/43 (4.7%)	7/19 (36.8%)	11.37 (1.85 to 126.32)	0.0024
Venous thrombosis	5/60 (8.3%)	7/34 (20.6%)	2.82 (0.7 to 12.38)	0.1117	2/43 (4.7%)	5/19 (26.3%)	7.04 (1.02 to 81.66)	0.0238
Peripheral pulmonary embolism	3/60 (5%)	1/34 (2.9%)	0.58 (0.01 to 7.55)	1	2/43 (4.7%)	1/19 (5.3%)	1.14 (0.02 to 23.16)	1
Hemofiltration	1/60 (1.7%)	12/34 (3.3%)	30.99 (4.15 to 1387.39)	<0.0001	0/43 (0%)	6/19 (31.6%)	Inf (3.27 to Inf)	0.0004
Ventilator-free days	25 (17.75–27)	13.5 (0.5–21)	9 (4–13)	<0.0001	24 (14–26.5)	16 (7–22.5)	5 (0–11)	0.0552
ICU-free days	22 (14.75–25)	6.5 (0–18.5)	9 (4 to 15)	0.0003	19 (8–24)	16 (3.5–24)	2 (−3 to 8)	0.4263
Hospital-free days	2 (0–12)	0 (0–4.75)	0 (0–3)	0.0298	2 (0–9.5)	0 (0–8)	0 (0 to 3)	0.2295
In-hospital mortality	0/60 (0%)	7/34 (20.6%)	Inf (2.9 to Inf)	0.0005	0/43 (0%)	3/19 (15.8%)	Inf (0.99 to Inf)	0.0256

Inf, infinitive means >4 at sofa score. mathematical correct is infinitive if it is >4. Binary data are presented as no./total no. (%) and continuous data as medians (IQR). Estimates are odds ratios for binary variables and estimated median differences for continuous variables. Differences between patients without and with platelet transfusion assessed by Fisher’s Exact Test for binary variables and Wilcoxon Rank Sum Test for continuous variables. PC, Platelet Concentrate; SOFA, Sequential Organ Failure Assessment Score.
